# Air pollution-induced placental alterations: an interplay of oxidative stress, epigenetics, and the aging phenotype?

**DOI:** 10.1186/s13148-019-0688-z

**Published:** 2019-09-17

**Authors:** N. D. Saenen, D. S. Martens, K. Y. Neven, R. Alfano, H. Bové, B. G. Janssen, H. A. Roels, M. Plusquin, K. Vrijens, T. S. Nawrot

**Affiliations:** 1 Centre for Environmental Sciences, Hasselt University, Hasselt, Belgium; 2 Department of Public Health and Primary Care, Leuven University, Leuven, Belgium

**Keywords:** Air pollution, DOHaD, Telomeres, Placenta, Aging, Epigenetics, Oxidative stress

## Abstract

According to the “Developmental Origins of Health and Disease” (DOHaD) concept, the early-life environment is a critical period for fetal programming. Given the epidemiological evidence that air pollution exposure during pregnancy adversely affects newborn outcomes such as birth weight and preterm birth, there is a need to pay attention to underlying modes of action to better understand not only these air pollution-induced early health effects but also its later-life consequences. In this review, we give an overview of air pollution-induced placental molecular alterations observed in the ENVIR*ON*AGE birth cohort and evaluate the existing evidence. In general, we showed that prenatal exposure to air pollution is associated with nitrosative stress and epigenetic alterations in the placenta. Adversely affected CpG targets were involved in cellular processes including DNA repair, circadian rhythm, and energy metabolism. For miRNA expression, specific air pollution exposure windows were associated with altered miR-20a, miR-21, miR-146a, and miR-222 expression. Early-life aging markers including telomere length and mitochondrial DNA content are associated with air pollution exposure during pregnancy. Previously, we proposed the air pollution-induced telomere-mitochondrial aging hypothesis with a direct link between telomeres and mitochondria. Here, we extend this view with a potential co-interaction of different biological mechanisms on the level of placental oxidative stress, epigenetics, aging, and energy metabolism. Investigating the placenta is an opportunity for future research as it may help to understand the fundamental biology underpinning the DOHaD concept through the interactions between the underlying modes of action, prenatal environment, and disease risk in later life. To prevent lasting consequences from early-life exposures of air pollution, policy makers should get a basic understanding of biomolecular consequences and transgenerational risks.

## Air pollution and the Developmental Origins of Health and Disease hypothesis

Air pollution is a global public health issue causing premature death and disease. It comprises different pollutants in gaseous (i.e., carbon oxides, nitrogen oxides, sulfur oxides, and ozone), volatile (i.e., ammonia, polycyclic aromatic hydrocarbons, and quinones), or particulate form (i.e., coarse, fine or ultrafine particles, and black carbon) derived from both natural and anthropogenic sources. In 2015, a component of air pollution, namely particulate matter smaller than 2.5 μm in aerodynamic diameter (PM_2.5_), was estimated to cause 4.2 million of deaths worldwide of which 202,000 children younger than 5 years [1]. Children are at higher risk of adverse health effects caused by air pollution, even at low levels, because their immune system and lungs are not fully developed, especially during *in utero* and early life [2].

Life *in utero* is considered a particularly sensitive period during which maternal exposure to unfavorable conditions may not only influence fetal development and induce adverse pregnancy outcomes but also have long-term effects influencing offspring susceptibility to diseases later in adulthood, as postulated by the Developmental Origins of Health and Disease (DOHaD) hypothesis [3, 4]. Substantial evidence associates exposure to air pollution during pregnancy with a range of adverse health outcomes at birth, including increased risk of low birth weight [5–7] and prematurity [6, 8], and also in adult life, such as cardiovascular disease [9], respiratory problems [10–12], and neurodevelopmental alterations [13] and even cancers [14]. However, the biological chain of events through which exposure to air pollution *in utero* influences an individual’s later-life health is still poorly known. As the placenta is a crucial organ for fetal development, alterations in the placenta on the molecular level, induced by air pollution, may be important as to the early origins of health and disease. This altered biomolecular functioning of the placenta may contribute to early and even later-life health consequences. In this review, we describe all the available evidence of placental molecular processes associated with prenatal air pollution exposure in the ENVIR*ON*AGE (ENVIRonmental influence *ON* AGEing in early life) birth cohort, situated in Belgium [15]. The biomolecular processes associated with air pollution exposure can be categorized into nitrosative stress, epigenetic alterations, and aging markers. Alterations in these placental molecular processes may lead to an altered newborn phenotype which may underlie a higher susceptibility for developing diseases later in life.

## Can air pollution particles reach the human placenta?

**Fig. 1:**
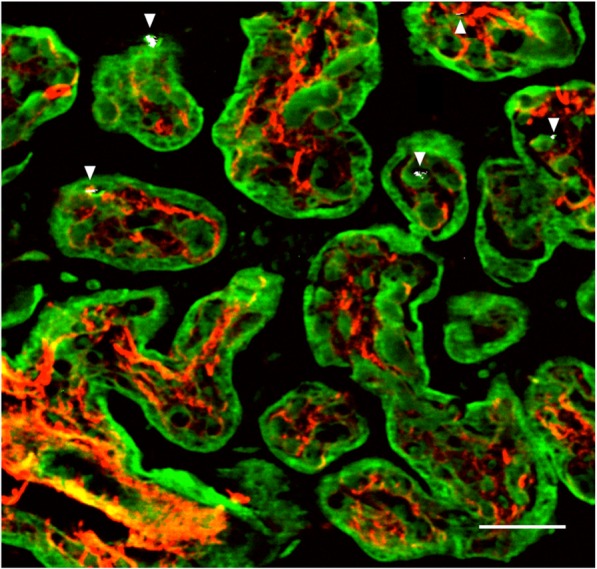
Evidence of black carbon particles from ambient air pollution in human placenta. White-light generation by the black carbon particles (white and further indicated using arrowheads) under femtosecond pulsed laser illumination is observed. Second harmonic generation from collagen (red) and two-photon autofluorescence from placental cells (green) are detected simultaneously. Scale bar 40 μm [18]

In a recent review, numerous investigations examined whether (nano) particles, in general, can pass the placenta and showed a dependency on size, shape, and surface charge [16]. Furthermore, a study by Valentino et al. [17] strengthened the hypothesis of transplacental particle translocation by showing “nanoparticle-like” aggregates in the cytoplasm of placental trophoblastic cells of rabbits exposed to aerosolized diesel exhaust particles. While these experimental studies show that translocation across the placenta is biologically possible, no such direct evidence in the context of human life exists. Recently, we detected the abundant presence of black carbon (BC) particles in human placenta at both the maternal and fetal side (Fig. 1) [18]. These findings confirm that ambient particles can be translocated directly towards the fetus and represent a potentially novel mechanism explaining the adverse effects from early life onwards, in addition to particle-induced inflammation in the lungs. Furthermore, we showed that urinary carbonaceous particles reflect residential BC exposure and traffic-related exposure [19], showing the translocation of particles from the lung to the system.

## Air pollution and placental oxidative/nitrosative stress

As air pollution particles may translocate into and cross the placental barrier [18, 20], they may induce placental modifications [21]. Oxidative stress may be one of the key elements of air pollution-induced placental alterations. Air pollution particles are able to generate reactive oxygen/nitrogen species (ROS/RNS) in both a direct and indirect way [22]. Particles may have free radicals present on their surface or may directly generate reactive hydroxyl radicals via a Fenton reaction in the presence of soluble transition metals on the particle surface, such as for instance iron [23]. One of the indirect sources of ROS production is through the PM-induced altered functioning of NADPH oxidases, telomere-mitochondrial dysregulation, and activation of inflammatory cells [22, 24]. These generated ROS can in turn directly induce lipid, protein, and DNA damage. Within the ENVIR*ON*AGE birth cohort, we assessed placental nitrosative stress and mitochondrial 8-deoxyguanosine damage. We observed that each interquartile range (IQR) increment in entire pregnancy PM_2.5_ exposure resulted in a 35.0% (95% CI 13.9 to 60.0%) increase in placental 3-nitrotyrosine levels, whereas an IQR increase in BC showed a 13.9% (95% CI − 0.21 to 29.9%) increase [25]. Direct oxidative damage in mitochondria was measured by 8-hydroxy-2′-deoxyguanosine (8-OHdG) levels in cord blood and maternal blood samples. Interestingly, we observed that elevated exposure to PM_10_ during trimester 1 and 2 of pregnancy resulted in an increase in mitochondrial 8-OHdG, while results for PM_2.5_ were less pronounced [26]. Whether this observation is reflective of placental mitochondrial damage remains to be elucidated. Within this regard, a study of 891 newborns of the Czech Republic showed increased 8-OHdG in placenta in association with exposure to PM_2.5_ in the first 4 months of pregnancy [27]. These authors also observed that newborns with above-median levels of 8-OHdG had higher probability of intrauterine growth restriction compared with newborns below-median level of oxidative DNA damage.

Taken together, these findings demonstrate that air pollution-related ROS/RNS production may affect the *in utero* environment. This can be supported by previous studies investigating smoking during pregnancy [28] and environmental toxic metals [29] in association with oxidative stress in the placenta.

## Air pollution-induced placental epigenetic alterations

Placental epigenetics is another important target to study early-life effects of air pollution, which includes changes in DNA methylation, histone and noncoding RNA modification, and chromatin remodeling. These processes are able to influence health outcomes during the life course and even across generations [30–32]. During pregnancy, especially DNA methylation is an important mechanism as it is involved in “epigenetic reprogramming”. During this process, DNA methylation patterns are erased and re-established, first in gametogenesis and again in early embryogenesis [33]. These epigenetic waves make the early embryonic development a critical period [34]. Within the ENVIR*ON*AGE birth cohort, we have studied placental epigenetic signatures in association to air pollution on different levels, from global DNA methylation, gene-specific DNA methylation to miRNA expression, and we focused both on nuclear and mitochondrial DNA (mtDNA) targets. Our findings from epigenetic alterations induced by air pollution exposure in the ENVIR*ON*AGE birth cohort are summarized in Table 1, in addition to other available evidence.

**Table 1:** Overview of placental epigenetic targets in association with air pollution exposure

Author	Study population	Technique	Studied air pollutant	Result
DNA methylation				
Global DNA methylation				
Janssen et al. [35]	240 mother-newborn pairs from the ENVIR*ON*AGE birth cohort, Belgium	UPLC/MS-MS	+ 5 μg/m^3^ PM_2.5_ during the implantation period (6–21 days after conception)	Global methylation − 1.08%, 95% CI − 1.80 to − 0.36%, *p* = 0.004
			+ 5 μg/m^3^ PM_2.5_ during the first trimester	Global methylation − 2.41%, 95% CI − 3.62 to − 1.20%, *p* = 0.0001
			+ 5 μg/m^3^ PM_2.5_ during the second trimester	Global methylation − 1.51%, 95% CI − 2.66 to − 0.36%, *p* = 0.01
			+ 5 μg/m^3^ PM_2.5_ during the entire pregnancy	Global methylation − 2.19%, 95% CI − 3.65 to − 0.73%, *p* = 0.004
Kingsley et al. [36]	471 mother-newborn pairs from the RICHS birth cohort, USA	Bisulfite-PCR-pyrosequencing	≤ 150 m primary highway or primary road or ≤ 50 m from secondary road	Living close to major roadway: *LINE-1* − 0.82%, 95% CI − 1.57 to − 0.07%, *p* = 0.03 *AluYb8*: no association, *p* = 0.07
Cai et al. [37]	181 mother-newborn pairs (80 fetal growth restriction newborns, 101 normal newborns) from Wenzhou, China	Bisulfite-PCR-pyrosequencing	+ 10 μg/m^3^ PM_10_ during the first trimester of pregnancy	Fetal growth restricted newborns: *LINE-1* − 1.78%, 95% CI − 3.35 to − 0.22% , *p* < 0.05 Normal newborns: *LINE-1* no association
Maghbooli Z et al. [38]	Nested case-control (*n* = 50/50) of pregnant women in Tehran, Iran	RP-HPLC	PM_2.5_ and PM_10_ during the first trimester	Global methylation: *r* = 0.26 (*p* = 0.01) and *r* = 0.38 (*p* = 0.0001) respectively
Abraham et al. [39]	668 mother-newborn pairs from the EDEN cohort, France	Bisulfite-PCR-pyrosequencing	+ 10 μg/m^3^ PM_10_ day before birth	Global methylation: *Alu*: *β* = 0.08; *p* = 0.01 *LINE-1*: *β* = 0.09; *p* = 0.28
Gene-specific methylation				
Janssen et al. [49]	381 mother-newborn pairs from the ENVIR*ON*AGE birth cohort, Belgium	Bisulfite-PCR-pyrosequencing	+ 7.8 μg/m^3^ PM_2.5_ during the first trimester of pregnancy	*MT-RNR1* + 1.27%, 95% CI 0.23 to 2.32%, *p* < 0.05 *D-loop* + 0.44%, 95% CI 0.12 to 0.75%, *p* < 0.05
			+ 3 μg/m^3^ PM_2.5_ during the entire pregnancy	*MT-RNR1* + 0.91%, 95% CI 0.56 to 4.18%, *p* < 0.05 *D-loop* + 0.21%, 95% CI − 0.003 to 1.02%, *p* > 0.05
Cai et al. [37]	181 mother-newborn pairs (80 fetal growth restriction newborns, 101 normal newborns) from Wenzhou, China	Bisulfite-PCR-pyrosequencing	+ 10 μg/m^3^ PM_10_ during the first trimester of pregnancy	Fetal growth restricted newborns: *HSD11B2* + 1.03%, 95% CI 0.07 to 1.98%, *p* < 0.05
			+ 10 μg/m^3^ PM_10_ during the second trimester of pregnancy	Fetal growth restricted newborns: *HSD11B2* + 2.23%, 95% CI 0.69 to 3.76%, *p* < 0.05 Total population: *HSD11B2* + 1.42%, 95% CI 0.24 to 2.57%, *p* < 0.05
			+ 10 μg/m^3^ PM_10_ during the entire pregnancy	Total population: *HSD11B2* + 1.98, 95% CI 0.53 to 3.43%, *p* < 0.05
Saenen et al. [48]	361 mother-newborn pairs from the ENVIR*ON*AGE birth cohort, Belgium	Bisulfite-PCR-pyrosequencing	+ 7.5 μg/m^3^ PM_2.5_ during the second trimester	*LEP* − 1.4%, 95% CI − 2.7 to − 0.19%, *p* = 0.02
Neven et al. [45]	463 mother-newborn pairs from the ENVIR*ON*AGE birth cohort, Belgium	Bisulfite-PCR-pyrosequencing	+ 3.84 μg/m^3^ PM_2.5_ during the entire pregnancy	*APEX1* + 7.34%, 95% CI 0.52 to 14.16%, *p* = 0.009 *OGG1* + 13.06%, 95% CI 3.88 to 22.24%, *p* = 0.005 *ERCC4* + 16.31%, 95% CI 5.43 to 27.18%, *p* = 0.003 *p53* + 10.60%, 95% CI 4.46 to 16.74%, *p* = 0.003 *DAPK1 −* 12.92%, 95% CI − 22.35 to − 3.49%, *p* = 0.007
			+ 0.36 μg/m^3^ BC during the entire pregnancy	*APEX1* + 9.16%, 95% CI 4.06 to 14.25%, *p* = 0.005 *ERCC4* + 27.56% 95% CI 17.58 to 37.55%, *p* < 0.0001
Nawrot et al. [42]	407 mother-newborn pairs from the ENVIR*ON*AGE birth cohort, Belgium	Bisulfite-PCR-pyrosequencing	+ 8.9 μg/m^3^ PM_2.5_ during the third trimester	Log(fold change) *NPAS2* 0.16, 95% CI 0.06 to 0.27, *p* = 0.002 Log(fold change) *CRY1-2* 0.59, 95% CI 0.22 to 0.95, *p* = 0.002 Log(fold change) *PER1* − 0.51, 95% CI − 0.90 to − 0.13, *p* = 0.001 Log(fold change) *PER3* 0.42, 95% CI 0.18 to 0.67, *p* = 0.001
			+ 7.9 μg/m^3^ PM_2.5_ during the first trimester	Log(fold change) *CLOCK* − 0.59, 95% CI − 0.93 to − 0.25, *p* < 0.001
miRNA expression				
Tsamou et al. [65]	210 mother-newborn pairs from the ENVIR*ON*AGE birth cohort, Belgium	qRT-PCR	+ 5 μg/m^3^ PM_2.5_ during the first trimester of pregnancy	*miR-20a* + 70.9%, 95% CI 16.7 to 150.3%, *p* = 0.007 *miR-21* + 73.7%, 95% CI 11.7 to 170.1%, *p* = 0.015
			+ 5 μg/m^3^ PM_2.5_ during the second trimester of pregnancy	*miR-146a* − 30.9%, 95% CI − 48.0 to − 8.1%, *p* = 0.012 *miR-222* − 25.4%, 95% CI − 43.0 to − 2.4%, *p* = 0.034 *miR-21* − 33.7%, 95% CI − 53.2 to − 6.2%, *p* = 0.022

### Placental global DNA methylation

In 2013, we were the first to show in 240 mother-newborn pairs that placental global DNA methylation was inversely associated with first trimester PM_2.5_ exposure, especially during the critical period of implantation [35]. These findings were confirmed by another study which showed that pregnant mothers living close to major roadways (i.e., a marker of traffic-related air pollution) had lower levels of placental DNA methylation in *LINE-1* but not *AluYb8*, which are surrogate markers of global DNA methylation [36]. Furthermore, in a Chinese study involving 181 mother-newborn pairs (80 fetal growth restriction newborns, 101 normal weight newborns), placental *LINE-1* DNA methylation was inversely associated with first trimester PM_10_ exposure [37]. In contrast, a nested case-control study (*n* = 100) in Iran showed a positive correlation of global methylation with first trimester PM_2.5_ and PM_10_ exposure [38]. It should be mentioned that the Iran study did not use the same technique nor investigated *LINE-1* DNA methylation. Furthermore, the EDEN cohort showed a positive association of placental *Alu* DNA methylation with day before birth PM_10_ exposure but not with placental *LINE-1* DNA methylation [39]. Nevertheless, these studies highlight that air pollution exposure already has an important impact on methylation patterns very early in embryonic development, directly after conception. This may be critical in development as it has been shown in mice that disturbances of DNA methylation in the placenta are associated with abnormal embryonic development [40] and that genetic inactivation of DNA methyltransferases (DNMTs) is lethal to developing mouse embryos [41].

### Placental candidate gene methylation

We have carried out different candidate gene methylation studies (Table 1). The rationale of these studies was based on the DOHaD hypothesis, in which we focused on key biological processes that are involved both in growth and development early in life and in age-related diseases later in life. We analyzed in the placentas of 407 newborns the promoter regions of regulatory genes in the circadian pathway (i.e., the central biological clock that maintains the daily cellular rhythm in accordance with the external environment). PM_2.5_ exposure during the last trimester of pregnancy was positively associated with placental methylation of *CLOCK*, *BMAL1*, *NPAS2*, *CRY1-2*, and *PER1-3* [42]. Previous findings stipulated that dysfunctions in the clock mechanism are prevalent in a variety of diseases, such as cancer, metabolic conditions, and neurological disorders [43, 44].

Further, we evaluated whether promoter regions of key DNA repair genes (including base-excision and nucleotide-excision repair genes) and tumor suppressor genes were differentially methylated in the placenta (*n* = 463). Higher entire pregnancy PM_2.5_ exposure was positively associated with methylation of the promoter regions from repair genes (*APEX1*, *OGG1*, *ERCC4*) and from the tumor suppressor *p53*, whereas promoter methylation of *DAPK1* was inversely associated. Similar findings were observed for *APEX1* and *ERCC1* in association with BC exposure [45]. In addition, we found that increased levels of both PM_2.5_ and BC were positively associated with higher mutation rates in placental DNA. These findings are in line with a study of Perera and colleagues [46], who showed that air pollution can induce aromatic DNA adducts in cord blood, and with an experimental study of Zhou and colleagues [47], in which hypermethylation of *p53* was shown in human bronchial epithelial cells after 10 days of PM_2.5_ exposure.

We also observed an inverse association between placental *LEP* promoter methylation (i.e., an energy-regulating hormone involved in fetal growth and development) and PM_2.5_ exposure during the second trimester of pregnancy [48]. Additionally, this association was strengthened by the determination of the oxidative/nitrosative stress biomarker 3-nitrotyrosine (3-NTp) [48], which showed a similar association as the modeled PM_2.5_ exposures, that was independent of maternal smoking.

Aside from the ENVIR*ON*AGE birth cohort, further evidence was found by Cai et al. [37] showing that exposure to PM_10_ during the first two trimesters of pregnancy was positively associated with placental methylation of *HSD11B2* (i.e., genes involved in the glucocorticoid metabolism and fetal growth). The observed associations were more pronounced in the fetal growth-restricted newborn subset (*n* = 80), compared to the normal growth newborns (*n* = 101).

Finally, we evaluated methylation of two regions of the mtDNA, i.e., D-loop control region and the 12S rRNA. In a study sample of 381 mother-newborn pairs, higher methylation levels of these two mitochondrial genome regions in association with prenatal PM_2.5_ exposure were observed, with the first trimester as most critical exposure window [49]. A higher methylation of mtDNA in relation to air pollution exposure is in accordance with a study in the blood of steelworkers [50].

Altogether, these candidate-based methylation studies show that mapping placental epigenome modifications attributable to air pollution offers a unique opportunity to unravel biomolecular signatures playing a potential role in the mediation of air pollution influence on postnatal life [51].

### Placental miRNA expression

In placental tissue, microRNA expression has been observed both in extracellular vesicles and in tissue biopsies. Several studies showed the ability of microRNA expression patterns to link pregnant women who were at risk of developing preeclampsia [52], preterm birth [53], or fetal growth restriction [54]. Furthermore, microRNA expression patterns in placental tissue have been shown to act in a sexually dimorphic manner in relation to both maternal obesity [55] and placental stress [56].

Until now, most studies focused on chemical exposures including cadmium [57], phthalates [58], arsenic [59], and endocrine disrupting chemicals [60] in association with placental miRNAs. Moreover, multiple studies confirm a role for miRNAs in the response to air pollution exposure in adults [61–63]. However, the literature on air pollution exposure in association with placental or, more broadly, early in life microRNA expression in humans is limited.

The first evidence for a role of miRNA expression in response to air pollution exposure in prenatal life came from an animal study [64]. In this study, pregnant rats were exposed to PM_2.5_ for extended periods of time, causing an increased number of immune cells in mother rats. Expression levels of cerebral cortical miR-6315, miR-3588, and miR-466b-5p were upregulated while a decreased expression of miR-338-5p and let-7e-5p was observed. Further, PM_2.5_ exposure increased miR-3560 and let-7b-5p in the hippocampus, while miR-99b-5p, miR-92b-5p, and miR-99a-5p were decreased. All of these miRNAs were related to neurobiological processes [64].

We were the first human cohort (ENVIR*ON*AGE) on air pollution exposure and placental miRNA expression in which we investigated six miRNAs (miR-16, miR-20a, miR-21, miR-34a, miR-146a, miR-222) in 210 placenta samples (Table 1). These miRNAs are involved in important cellular processes such as cell cycle, proliferation, apoptosis, inflammation, and angiogenesis. A positive association with first-trimester PM_2.5_ exposure was identified for placental miR-20a expression, whereas second-trimester exposure was negatively associated with the expression of miR-21a, miR-146a, and miR-222. Furthermore, first-trimester PM_2.5_ exposure was positively associated with miR-21 expression, whereas it was negatively associated with second-trimester PM_2.5_ exposure. Tumor suppressor phosphatase and tensin homolog (PTEN) was identified as a common target of the miRNAs significantly associated with PM exposure [65].

One other study used cord blood as biological sample for miRNA expression and smoking status as exposure. They investigated whether miR-155 and miR-233 expression in 450 cord blood and maternal blood samples from the LINA (Lifestyle and Environmental Factors and Their Influence on Newborns Allergy Risk) study was associated with smoking behavior during pregnancy [66]. They found that increased maternal urinary cotinine concentrations (i.e., a marker for short-term smoking exposure) during pregnancy were associated with elevated miR-223 expression in cord blood. Cord blood miR-155 expression was related to lower toluene metabolite S-benzylmercapturic acid concentrations in maternal urine. Moreover, they demonstrated in newborns that a high miR-223 expression in cord blood cells was associated with lower cord blood regulatory T cell numbers.

## Air pollution exposure and the aging phenotype

Besides epigenetic alterations in association with air pollution exposure, more downstream placental targets to evaluate the potential impact of air pollution in the DOHaD hypothesis were evaluated in the ENVIR*ON*AGE birth cohort. We have a profound interest in aging-related targets, including telomeres and mitochondria. This is because of the fact that these targets have been widely studied in adult populations and have been associated with age-related diseases. Telomeres shorten throughout the life-span, and this shortening may be influenced by environmental factors, including air pollution [67]. Telomeres play a role in cell senescence and human aging and are indicative of disease risks, and in this regard, short telomeres have independently of chronological age been associated with higher risks for cardiovascular disease [68], type 2 diabetes [69], respiratory diseases [70], and mortality [71]. In addition, mitochondrial dysfunction and mutations play an import role in neurodegenerative diseases [72], cardiovascular diseases [73], and aging [74].

In the ENVIR*ON*AGE birth cohort, we observed for 174 newborns a decrease of 17.4% in placental mtDNA content for a 10-μg/m^3^ increment in PM_10_ exposure during the third trimester of pregnancy [75]. Furthermore, in a larger subset of 381 individuals, we observed that an increment of 10-μg/m^3^ in PM_2.5_ during the third trimester was associated with a decrease of 23.6% in mtDNA content [49]. In 2017, we showed that placental telomere length (TL) was negatively associated with PM_2.5_ exposure during weeks 15–27 of gestation [76]. In this study, we applied a distributed lag model which enabled us to investigate weekly exposures during pregnancy in association with placental TL. The estimated effect of a 5-μg/m^3^ increase in PM_2.5_ during the second trimester and entire pregnancy was associated with 7.1% and 13.2% shorter placental TL, respectively. Furthermore, shorter placental TL has been observed with increased residential proximity to a major road and a decreased residential greenness [77], and with prenatal cadmium exposure [78].

The importance of placental TL for later-life conditions is rather unclear. However, if placental TL relates to cell senescence, this may influence placental aging, with health consequences on the short- and potential long-term. In this regard, it has been shown that placental senescence is observed in placentas complicated with intrauterine growth restriction or preeclampsia, and indeed, shorter placental TL was observed in these conditions [78]. This directly impacts fetal development and outcomes. In uncomplicated pregnancies, a high variability in placental TL is observed [79], and although these pregnancies may result in a healthy newborn phenotype, later-life consequences may be programmed at the level of telomeres. Indeed, it has been shown that placental TL may predict later-life TL [80], and therefore, changes in placental TL may be predictive for later-life risks in telomere length-associated diseases. However, prospective follow-up studies are needed to confirm whether newborn TL indeed reflects later-life disease risks.

## Interplay of oxidative stress, epigenetics, and the air pollution-induced telomere/mitochondrial axis of aging

**Fig. 2:**
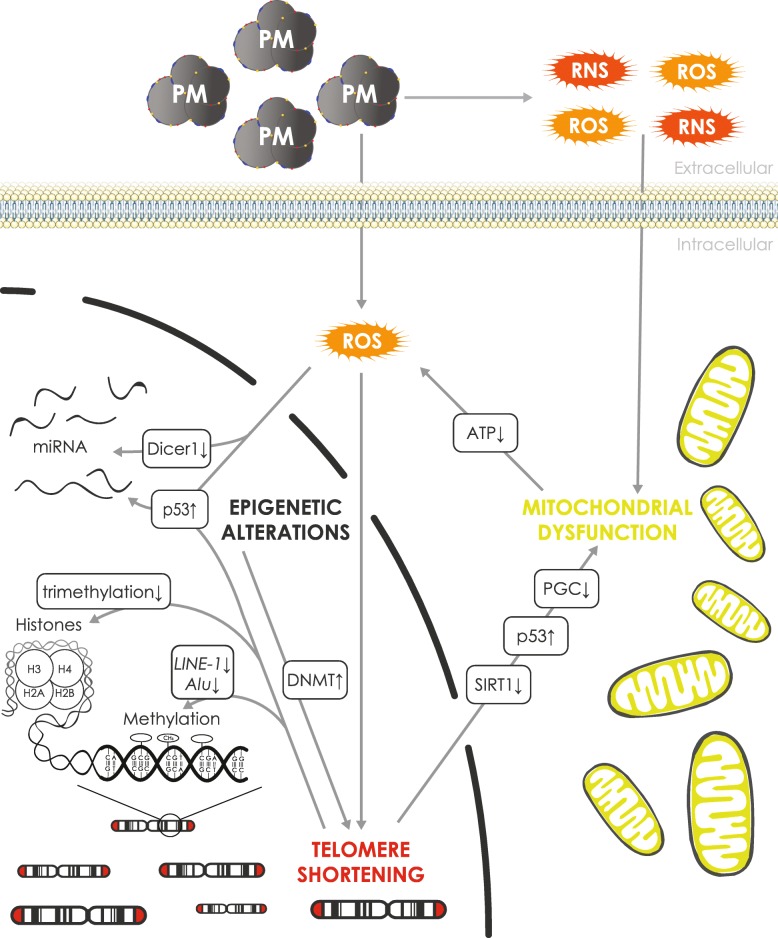
An extended view of the air pollution-induced telomere-mitochondrial aging hypothesis. Our previous hypothesis showed that the presence of air pollution-induced ROS within cells induces DNA damage which leads to telomere shortening. Both DNA damage and telomere shortening are associated with increased levels of p53, which on its turn leads to increased mitochondrial dysfunction. Furthermore, disturbances in mitochondria can also increase cellular ROS production. We extended this view with epigenetic regulation. A dynamic regulation exists between epigenetic marks and TL. High trimethylated histones at the subtelomeric and telomeric region as well as high subtelomeric DNA methylation by DNMTs are a negative regulator of TL. Additionally, shortening of telomeres leads to a decrease in both histone trimethylation and subtelomeric DNA methylation and global DNA methylation (*Alu*, *LINE-1*). Furthermore, microRNAs might be involved through *DICER1* regulation that is linked to DNMT expression and on its turn affects methylation processes of the genome and subtelomeric regions. Finally, miRNAs are also under the regulation of both DNA methylation and p53. Both p53 and *DICER1* may be under regulation of ROS

We previously proposed the air pollution-induced telomere-mitochondrial aging hypothesis [24], with its fundamental basis on findings of a direct link between mitochondria and telomeres [81, 82]. Telomeres are highly sensitive to ROS, and air pollution has shown to increase levels of ROS, which may target telomeres, and shorten them leading to potential dysfunctional telomeres (Fig. 2). Telomere dysfunction in mice showed p53 activation which resulted in suppression of peroxisome proliferator-activated receptor gamma (*Pparγ*) co-activator 1 alpha and beta (*Pgc-1α,β*) genes [81]. Repression of *Pgc-1α,β* leads to a strong decrease in mitochondrial biogenesis and function, subsequently leading to an impaired ATP generation and an increase in ROS production. Alterations in the energy metabolism are a driver of the aging process. Furthermore, DNA damage at telomeres activates several signaling pathways and reduces *Sirt1* gene expression, which leads to mitochondrial dysfunction, partially through elevated p53 and reduced PGC [83]. This indicates an intimate relationship and interaction between telomeres and mitochondria.

However, extending this view with epigenetic regulation of TL and mitochondria may be essential in understanding air pollution-induced placental molecular alterations as shown in the ENVIR*ON*AGE study (Fig. 2). In this regard, clear evidence is available that a dynamic regulation of epigenetic marks and TL is present, because both epigenetic marks may influence TL regulation and homeostasis, but vice versa telomere shortening may alter epigenetic marks. In this regard, it has been shown that telomeric and subtelomeric regions are enriched in trimethylated histones H3K9me3 and H4K20me3 (trimethylation of histone H3 at lysine 9 and of histone H4 at lysine 20), and subtelomeric regions are highly methylated by DNMT1, DNMT3a, and DNMT3b enzymes [84]. This high DNA and histone methylation state has shown to be a negative regulator of TL [84], as cells deficient in DNMTs displayed a strong decrease of subtelomeric DNA methylation and showed elongated telomeres, potentially due to telomerase, and increase telomere recombination [85]. On the other hand, TL may influence the epigenetic landscape. As telomeres shorten, this may lead to a decrease in trimethylation of H3K9 and H4K20 in the telomeric and subtelomeric region as well as a decrease in subtelomeric DNA methylation as shown in telomerase-deficient Terc^−/−^ mice experiments with short telomeres [86]. However, subsequently, this may lead to telomere elongation and maintenance processes as described above. In human population-based studies, lower global DNA methylation (*LINE-1* and *Alu*) has been associated with shorter telomeres [87, 88].

Within the context of air pollution actions on the epigenetic landscape, several theories exist how changes in DNA methylation status can be induced: (1) ROS generated by air pollution-induced oxidative stress can react with DNA, resulting into different DNA lesions, including base modifications, strand breaks, and inter- and intra-strand crosslinks [89]. Due to these DNA alterations, DNMTs are not able to recognize this damaged DNA as a reaction substrate, which leads to a global hypomethylation [90]. Additionally, it has been shown that the repair of damaged DNA by homologous recombination (HR) induces DNA methylation [91], and chromatin, damaged by oxidative stress, recruits DNMT1, which results in DNA methylation changes [92]. One of the most frequently occurring ROS-generated DNA lesions is the oxidation of guanine, resulting in the formation of 8-OHdG [93]. The presence of an 8-OHdG residue inhibits the ability of DNMT to methylate nearby located cytosines [94]. Furthermore, ROS can increase 5mC oxidation via 5hMC, which eventually leads to global hypomethylation [95]. (2) Environmental chemicals may interfere with S-adenosyl methionine (SAM) which results in a methylation reduction by DNMT due to a depletion of available methyl groups from SAM [96, 97]. Furthermore, *DNMT* gene expression is reduced by long-term environmental exposures [98, 99]. (3) Another suggested mechanism is the so-called transcription factor occupancy theory, in which an interplay between the presence or absence of transcription factors (TF) and the degree of gene-specific DNA methylation exists [96]. In this regard, research by Martin and Fry [100] showed that genes of which the methylation status (evaluated in cord blood or placenta) was associated with prenatal exposures (including arsenic, cadmium, lead, manganese, mercury, and tobacco smoke), shared binding sites for TFs that had a known relationship with these prenatal exposures.

A role of microRNAs in the regulation of DNA methylation and telomeres has also been shown (Fig. 2). In *Dicer1*-deficient mice, a downregulation of the miR-290 cluster was observed. This downregulation leads to an increase in mRNA levels of *Rbl2* (retinoblastoma-like 2 protein) that subsequently inhibits DNMT expression. This decrease in DNMT results in a hypomethylation of the genome and subtelomeric regions, leading to the aforementioned increase in TL and telomere recombination [101]. In this regard, we could evaluate in a small sub-population of the ENVIR*ON*AGE birth cohort that placental TL was associated with miRNA expression. More precisely, we observed that miR-34a, miR-146a, miR-210, and miR-222 expression was positively associated with placental TL in newborn girls [102]. However, in this small sub-population (*n* = 203), the mediating effect of miRNA expression in the association between air pollution and TL could not be evaluated. Nevertheless, as high exposure to PM_2.5_ during second trimester was associated with both shorter placental TL and a reduced miR-146a and miR-222 expression, and both miRNAs were associated with longer placental TL in girls, these miRNA targets may warrant further attention. Also, in these miRNA-air pollution associations, ROS may play an important role [103]. First, ROS could act on the biogenesis enzymes of miRNAs, and it has been shown that H_2_O_2_-treated JAR trophoblast cells selectively inhibited Dicer activity [104, 105]. Second, ROS may regulate miRNA expression through the alteration of transcription factors, including p53 and NF-κB [103]. p53 is a major tumor suppressor involved in cellular senescence and is assumed to play a pivotal role in our proposed “hypothesis” [24]. Air pollution exposure may alter the expression of p53, and recent studies showed that p53, as a transcription factor, is an important regulator of miRNA expression [106]. Therefore, p53 may be an important target which links air pollution and miRNA regulation with a potential implication as to the aging phenotype and later-life diseases. Finally, miRNA genes are by themselves under the regulation of DNA methylation [103], and therefore, the air pollution/ROS-related DNA methylation regulatory mechanisms may affect miRNA expression, indicating a close interplay between the different epigenetic mechanisms.

Aside from nuclear DNA, it should be noted that mitochondria are the second cellular location to house an abundance of DNA (mtDNA). During recent years, it has been shown that the mitochondrial genome can also undergo epigenetic modifications. Within the ENVIR*ON*AGE birth cohort, we were able to evaluate that indeed an important interplay exists between placental mtDNA content and mtDNA methylation. We observed that the effect of prenatal PM_2.5_ exposure on placental mtDNA content was mediated for 54% by mitochondrial 12S RNA methylation and for 27% by mitochondrial D-loop methylation [49]. Although studies on mitochondrial epigenetics are still in its infancy, PM_2.5_ exposure may be a potential candidate with important links to mitochondrial epigenetics [107].

## Challenges and opportunities for using the placenta in early-life environmental exposure research

The findings we reviewed in this paper underscore the sensitivity of the biomolecular system to environmental factors during the early period of developmental plasticity. Methylation patterns are re-established during early pregnancy, making this a highly sensitive window of susceptibility to the effects of prenatal air pollution exposure. This may lead to an adaptive response altering placental and fetal development with possibly a long-lasting impact in later life. However, the crucial question remains about the time window in which air pollution exposure influences biomolecular processes during pregnancy as measurements on placenta can only be performed at birth. In this respect, it is noteworthy that in observational studies, the conventional approach of averaging exposures over relatively large time windows (trimesters or the entire pregnancy) can be further refined by using distributed lag models to allow a more detailed investigation of prenatal exposure windows and enable the identification of critical periods during pregnancy for the association with air pollution exposure [76, 108].

Until now, most studies investigating early-life air pollution exposure and placental alterations have focused on DNA methylation, while we were the first investigating microRNAs and even aging-related markers. This can probably be explained by the interpretation capability and the availability of high-throughput laboratory techniques [109]. But even for biomolecular processes, changes need to be interpreted in the context of their biological relevance. For example, although DNA methylation is usually associated with alterations in gene expression [110], it is not known whether small changes in the methylation status of a given promoter necessarily translate into an alteration in gene expression [111, 112]. Furthermore, establishing a cutoff value for differential DNA methylation as biologically relevant is difficult, as this can depend on the type of study, sample size, heterogeneity of the tissue, the method or technique used, or even interpretation of the data. In this respect, larger differences are desirable between cases and controls for a certain disease phenotype, whereas for epidemiological studies, subtle changes in DNA methylation levels can have a functional meaning by revealing biological pathways involved in disease development or to unravel underlying mechanisms of action. In the ENVIR*ON*AGE birth cohort, the reported associations of air pollution exposure with biomolecular processes are generally low in terms of the size of the estimate, although they are significant in terms of nominal *p* values [113]. To prevail false-positive or false-negative findings, studies should participate in consortia to analyze their findings to improve the generalization of the results [30]. Within this context, the Pregnancy and Childhood Epigenetics cohort (PACE [114], *n* > 29,000) investigates the cord blood methylome. Similar approaches for placenta epigenetics are currently initiated but must carefully consider differences in the definition of exposures, biological sampling, laboratory techniques, and demographic and lifestyle characteristics of the study population.

Another challenge is that each molecular layer will not only interact with themselves but also display reciprocal relations with other biological networks as discussed in this review. Therefore, studies would benefit from using multi-omics approaches in which different molecular levels are integrated. So far, a few studies in adults included more than one molecular layer of epigenetics or other omics, for example by combining DNA methylation with the transcriptome [115], with inflammatory protein levels [115, 116], or with genetic variation [117].

In addition, the heterogeneity of the biological sample is also challenging when using the placenta. Gene regulation is tissue-, cell-, and context-specific, giving rise to cell-to-cell variation. Cellular composition explains a large part of the observed variability in gene regulation; thus, failing to account for the cellular heterogeneity may result in false-positive outcomes [109]. Measuring the cellular composition would be ideal but is in practice not always feasible. In the past years, efforts have been made to establish algorithms that predict peripheral and cord blood cell composition [118, 119]; however, for placenta, this is not available. Expanding these algorithms to underexplored tissues such as placenta will improve interpretation of results with regard to environmental exposures on placental molecular alterations.

Although molecular alterations are at times an adaptive reaction rather than a cause of disease, we now know that these changes may play an important role in diseases, including cancer, and occur long before disease develops. Epidemiological evidence demonstrated the role of diet and stress in changing the epigenetic program over generations. Examples of this are evidenced in the Dutch hunger winter study [120] and Holocaust survivors and their offspring [121]. These extreme living conditions entailed permanent changes in the epigenetic make-up, and among similar lines, evidence is accumulating that this occurs also for less stringent environmental conditions or exposures. Epigenetic analysis, as demonstrated in our overview, can be used to assess in utero and transgenerational effects. Therefore, epigenetics can lead us to understand the fundamental biology underpinning Barker’s DOHaD hypothesis in terms of interactions between the genome, prenatal environment, and disease risk. For example, Janssen et al. [35] showed placental hypomethylation in association with prenatal PM exposure. Furthermore, the observations of transplacental carcinogenesis by air pollution-induced *ALU* mutation rate parallels changes in the methylation of genes involved in the DNA repair machinery [45].

While we are only at the beginning to understand transplacental mechanisms, the evidence is mounting that prenatal exposure to ambient air pollution, oxidative stress, epigenetic alterations in DNA repair genes, metabolic genes, and changes in biological aging processes are all molecular processes involved in age-related diseases including cancer. The strength of molecular epidemiology within environmental health is the progress it offers in the understanding of fetal programming and the unraveling of the complex interplay between external and biological factors in order to increase our knowledge about DOHaD in terms of diseases at older age.

Developmental vulnerability should be a priority for environmental public health policies and practices to protect the most susceptible period of human life due to the long-term consequences. Follow-up of child or birth cohorts is crucial to understand the clinical consequences of early-life epigenetic changes on sub-optimal organ development resulting in a decreased reserve capacity of different organ systems and its risk later in life. The strategy aiming at effective protection of pregnant women, unborn children, and infants against lifelong consequences of exposure to combinations of adverse lifestyle factors requires that public health policy makers should get a basic understanding of epigenetic consequences and transgenerational risks.

## Data Availability

Data sharing is not applicable to this article as no datasets were generated or analyzed during the current study.
